# *N*-(Naphthalen-1-yl)benzamide

**DOI:** 10.1107/S1600536811045946

**Published:** 2011-11-05

**Authors:** Ruitao Zhu, Yuehong Ren, Wenjuan Li

**Affiliations:** a Department of Chemistry, Taiyuan Normal University, Taiyuan 030031, People’s Republic of China

## Abstract

In the title compound, C_17_H_13_NO, the N—H and C=O bonds are *anti* with respect to each other. The dihedral angle between the naphthalene ring system and the phenyl ring is 86.63 (5)°. In the crystal, N—H⋯O hydrogen bonds link mol­ecules into chains along [010].

## Related literature

For a related structure, see: Zhang *et al.* (2011 ▶). For standard bond-length data, see: Allen *et al.* (1987 ▶).
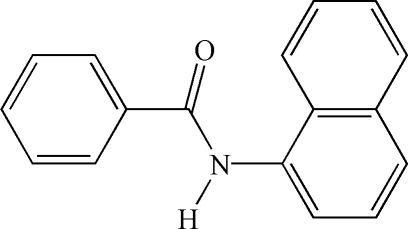


## Experimental

### 

#### Crystal data

C_17_H_13_NO
*M* *_r_* = 247.28
Orthorhombic, 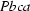

*a* = 8.2630 (8) Å
*b* = 9.3792 (9) Å
*c* = 33.806 (3) Å
*V* = 2620.0 (4) Å^3^
*Z* = 8
Mo *K*α radiation
μ = 0.08 mm^−1^
*T* = 298 K
0.45 × 0.24 × 0.13 mm

#### Data collection

Bruker SMART CCD diffractometer
Absorption correction: multi-scan (*SADABS*; Bruker, 2007 ▶) *T*_min_ = 0.966, *T*_max_ = 0.990
12210 measured reflections
2307 independent reflections
1253 reflections with *I* > 2σ(*I*)
*R*_int_ = 0.086

#### Refinement

*R*[*F* ^2^ > 2σ(*F* ^2^)] = 0.056
*wR*(*F* ^2^) = 0.129
*S* = 1.04
2307 reflections
172 parameters
H-atom parameters constrained
Δρ_max_ = 0.17 e Å^−3^
Δρ_min_ = −0.18 e Å^−3^

### 

Data collection: *SMART* (Bruker, 2007 ▶); cell refinement: *SAINT* (Bruker, 2007 ▶); data reduction: *SAINT*; program(s) used to solve structure: *SHELXS97* (Sheldrick, 2008 ▶); program(s) used to refine structure: *SHELXL97* (Sheldrick, 2008 ▶); molecular graphics: *SHELXTL* (Sheldrick, 2008 ▶); software used to prepare material for publication: *SHELXTL*.

## Supplementary Material

Crystal structure: contains datablock(s) I, global. DOI: 10.1107/S1600536811045946/lh5366sup1.cif

Structure factors: contains datablock(s) I. DOI: 10.1107/S1600536811045946/lh5366Isup2.hkl

Supplementary material file. DOI: 10.1107/S1600536811045946/lh5366Isup3.cml

Additional supplementary materials:  crystallographic information; 3D view; checkCIF report

## Figures and Tables

**Table 1:** Hydrogen-bond geometry (Å, °)

*D*—H⋯*A*	*D*—H	H⋯*A*	*D*⋯*A*	*D*—H⋯*A*
N1—H1⋯O1^i^	0.86	2.21	2.892 (3)	136

Symmetry code: (i) 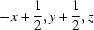.

**Fig. 1.**
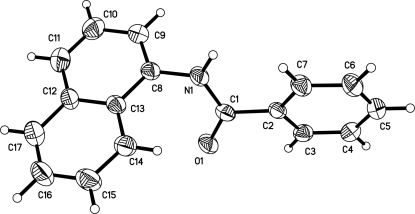
The molecular structure of the title compound with displacement ellipsoids drawn at the 30% probability level.

**Fig. 2.**
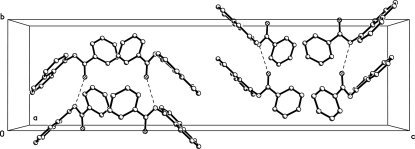
Part of the crystal structure of (I) with the donor-acceptor distances of hydrogen bonds drawn as dashed lines. H atoms are not shown.

## supplementary crystallographic information

### Comment

We recently determined the crystal structure of N-(1-naphthyl)benzenesulfonamide (Zhang *et al.*, 2011) and in this paper we present the crystal structure of the title compound (I).

The molecular structure of (I) is shown in Fig. 1. The bond lengths (Allen *et al.*, 1987) and angles are normal. The dihedral angle between the naphthylene ring system and the phenyl ring is 86.63 (5)°. In the crystal, N—H···O hydrogen bonds link molecules into chains along [010] (Fig. 2).

### Experimental

To a 100 ml round flask fitted with a condenser was added 1-naphthylamine (1.43 g, 10 mmol), dichloromethane (15 ml) and triethylamine(0.5 ml) with magnetic stirring. Benzoyl chloride (1.16 ml, 10 mmol) was added gradually. The reaction mixture was stirred at room temperature for 1 h and then refluxed for 2 h. The product precipitated as a white powder, which was washed three times with water and dichloromethane. Recrystallization from ethyl alcohol produced the crystals of the title compound.

### Refinement

H atoms were placed in idealized positions and allowed to ride on their respective parent atoms, with C—H = 0.93 Å, N—H = 0.86Å and *U*_iso_(H)= 1.2*U*_eq_(C,*N*).

### Crystal data

**Table:**

C_17_H_13_NO	*F*(000) = 1040
*M**_r_* = 247.28	*D*_x_ = 1.254 Mg m^−^^3^
Orthorhombic, *P**b**c**a*	Mo *K*α radiation, λ = 0.71073 Å
Hall symbol: -P 2ac 2ab	Cell parameters from 1469 reflections
*a* = 8.2630 (8) Å	θ = 2.7–20.5°
*b* = 9.3792 (9) Å	µ = 0.08 mm^−^^1^
*c* = 33.806 (3) Å	*T* = 298 K
*V* = 2620.0 (4) Å^3^	Prism, colorless
*Z* = 8	0.45 × 0.24 × 0.13 mm

### Data collection

**Table:**

Bruker SMART CCD diffractometer	2307 independent reflections
Radiation source: fine-focus sealed tube	1253 reflections with *I* > 2σ(*I*)
graphite	*R*_int_ = 0.086
φ and ω scans	θ_max_ = 25.0°, θ_min_ = 2.7°
Absorption correction: multi-scan (*SADABS*; Bruker, 2007)	*h* = −9→8
*T*_min_ = 0.966, *T*_max_ = 0.990	*k* = −10→11
12210 measured reflections	*l* = −40→26

### Refinement

**Table:**

Refinement on *F*^2^	Primary atom site location: structure-invariant direct methods
Least-squares matrix: full	Secondary atom site location: difference Fourier map
*R*[*F*^2^ > 2σ(*F*^2^)] = 0.056	Hydrogen site location: inferred from neighbouring sites
*wR*(*F*^2^) = 0.129	H-atom parameters constrained
*S* = 1.04	*w* = 1/[σ^2^(*F*_o_^2^) + (0.0469*P*)^2^] where *P* = (*F*_o_^2^ + 2*F*_c_^2^)/3
2307 reflections	(Δ/σ)_max_ < 0.001
172 parameters	Δρ_max_ = 0.17 e Å^−^^3^
0 restraints	Δρ_min_ = −0.18 e Å^−^^3^

### Special details

**Table:**

Geometry. All e.s.d.'s (except the e.s.d. in the dihedral angle between two l.s. planes) are estimated using the full covariance matrix. The cell e.s.d.'s are taken into account individually in the estimation of e.s.d.'s in distances, angles and torsion angles; correlations between e.s.d.'s in cell parameters are only used when they are defined by crystal symmetry. An approximate (isotropic) treatment of cell e.s.d.'s is used for estimating e.s.d.'s involving l.s. planes.
Refinement. Refinement of *F*^2^ against ALL reflections. The weighted *R*-factor *wR* and goodness of fit *S* are based on *F*^2^, conventional *R*-factors *R* are based on *F*, with *F* set to zero for negative *F*^2^. The threshold expression of *F*^2^ > σ(*F*^2^) is used only for calculating *R*-factors(gt) *etc*. and is not relevant to the choice of reflections for refinement. *R*-factors based on *F*^2^ are statistically about twice as large as those based on *F*, and *R*- factors based on ALL data will be even larger.

### Fractional atomic coordinates and isotropic or equivalent isotropic displacement parameters (Å^2^)

**Table:**

	*x*	*y*	*z*	*U*_iso_*/*U*_eq_	
N1	0.2455 (2)	0.6961 (2)	0.36346 (6)	0.0567 (6)	
H1	0.2671	0.7855	0.3655	0.068*	
O1	0.3279 (2)	0.48418 (17)	0.33881 (5)	0.0644 (5)	
C1	0.3422 (3)	0.6140 (3)	0.34048 (7)	0.0511 (7)	
C2	0.4657 (3)	0.6894 (3)	0.31610 (7)	0.0439 (6)	
C3	0.5181 (3)	0.6226 (3)	0.28213 (8)	0.0538 (7)	
H3	0.4779	0.5330	0.2756	0.065*	
C4	0.6293 (3)	0.6871 (3)	0.25781 (8)	0.0650 (8)	
H4	0.6620	0.6423	0.2346	0.078*	
C5	0.6924 (3)	0.8183 (3)	0.26772 (9)	0.0701 (8)	
H5	0.7681	0.8617	0.2513	0.084*	
C6	0.6433 (3)	0.8845 (3)	0.30176 (9)	0.0717 (8)	
H6	0.6874	0.9722	0.3087	0.086*	
C7	0.5284 (3)	0.8213 (3)	0.32578 (8)	0.0602 (7)	
H7	0.4933	0.8677	0.3485	0.072*	
C8	0.1091 (3)	0.6407 (2)	0.38450 (7)	0.0520 (7)	
C9	−0.0404 (4)	0.6895 (3)	0.37506 (8)	0.0648 (8)	
H9	−0.0521	0.7592	0.3557	0.078*	
C10	−0.1779 (3)	0.6353 (3)	0.39436 (9)	0.0813 (9)	
H10	−0.2800	0.6700	0.3880	0.098*	
C11	−0.1619 (4)	0.5323 (3)	0.42238 (9)	0.0821 (9)	
H11	−0.2536	0.4944	0.4344	0.099*	
C12	−0.0082 (3)	0.4827 (3)	0.43327 (8)	0.0628 (8)	
C13	0.1314 (3)	0.5404 (2)	0.41501 (7)	0.0516 (7)	
C14	0.2849 (3)	0.4941 (3)	0.42846 (8)	0.0630 (8)	
H14	0.3783	0.5321	0.4173	0.076*	
C15	0.2966 (4)	0.3944 (3)	0.45750 (9)	0.0806 (9)	
H15	0.3983	0.3662	0.4663	0.097*	
C16	0.1598 (5)	0.3343 (4)	0.47420 (9)	0.0965 (11)	
H16	0.1698	0.2635	0.4933	0.116*	
C17	0.0127 (5)	0.3784 (3)	0.46272 (9)	0.0897 (10)	
H17	−0.0783	0.3388	0.4746	0.108*	

### Atomic displacement parameters (Å^2^)

**Table:**

	*U*^11^	*U*^22^	*U*^33^	*U*^12^	*U*^13^	*U*^23^
N1	0.0702 (14)	0.0349 (11)	0.0651 (14)	−0.0063 (11)	0.0122 (12)	0.0000 (11)
O1	0.0830 (14)	0.0361 (10)	0.0742 (13)	−0.0041 (9)	0.0132 (10)	0.0025 (10)
C1	0.0635 (18)	0.0372 (15)	0.0526 (16)	0.0037 (14)	−0.0026 (14)	0.0038 (14)
C2	0.0440 (14)	0.0370 (14)	0.0507 (15)	0.0007 (12)	−0.0028 (12)	0.0061 (14)
C3	0.0499 (16)	0.0433 (16)	0.0681 (18)	0.0021 (12)	−0.0011 (14)	−0.0042 (15)
C4	0.0602 (17)	0.0634 (19)	0.0714 (19)	0.0100 (15)	0.0134 (15)	0.0011 (17)
C5	0.0666 (19)	0.0567 (19)	0.087 (2)	−0.0005 (16)	0.0210 (16)	0.0140 (18)
C6	0.076 (2)	0.0451 (16)	0.094 (2)	−0.0135 (15)	0.0137 (17)	−0.0008 (18)
C7	0.0731 (18)	0.0453 (16)	0.0621 (17)	−0.0050 (14)	0.0059 (14)	−0.0019 (15)
C8	0.0624 (18)	0.0399 (14)	0.0538 (16)	0.0019 (13)	0.0041 (14)	−0.0017 (14)
C9	0.0682 (19)	0.0570 (17)	0.0691 (19)	0.0079 (16)	0.0035 (16)	0.0083 (15)
C10	0.061 (2)	0.096 (2)	0.088 (2)	0.0123 (18)	0.0057 (17)	0.006 (2)
C11	0.072 (2)	0.090 (2)	0.084 (2)	−0.0040 (19)	0.0180 (17)	0.012 (2)
C12	0.078 (2)	0.0556 (17)	0.0551 (17)	0.0001 (15)	0.0143 (15)	0.0084 (15)
C13	0.0720 (19)	0.0381 (14)	0.0448 (14)	0.0031 (14)	0.0037 (14)	−0.0027 (13)
C14	0.0745 (19)	0.0567 (18)	0.0577 (18)	0.0079 (15)	0.0008 (15)	0.0013 (15)
C15	0.103 (3)	0.076 (2)	0.0631 (19)	0.0211 (19)	−0.0074 (18)	0.0149 (19)
C16	0.138 (3)	0.085 (2)	0.067 (2)	0.024 (3)	0.015 (2)	0.028 (2)
C17	0.109 (3)	0.083 (2)	0.077 (2)	0.005 (2)	0.0259 (19)	0.020 (2)

### Geometric parameters (Å, °)

**Table:**

N1—C1	1.354 (3)	C8—C13	1.408 (3)
N1—C8	1.431 (3)	C9—C10	1.405 (4)
N1—H1	0.8600	C9—H9	0.9300
O1—C1	1.225 (2)	C10—C11	1.360 (4)
C1—C2	1.490 (3)	C10—H10	0.9300
C2—C3	1.378 (3)	C11—C12	1.402 (4)
C2—C7	1.381 (3)	C11—H11	0.9300
C3—C4	1.373 (3)	C12—C17	1.406 (4)
C3—H3	0.9300	C12—C13	1.415 (3)
C4—C5	1.377 (3)	C13—C14	1.416 (3)
C4—H4	0.9300	C14—C15	1.359 (3)
C5—C6	1.369 (3)	C14—H14	0.9300
C5—H5	0.9300	C15—C16	1.384 (4)
C6—C7	1.383 (3)	C15—H15	0.9300
C6—H6	0.9300	C16—C17	1.341 (4)
C7—H7	0.9300	C16—H16	0.9300
C8—C9	1.356 (3)	C17—H17	0.9300
			
C1—N1—C8	122.9 (2)	C8—C9—C10	120.3 (3)
C1—N1—H1	118.5	C8—C9—H9	119.8
C8—N1—H1	118.5	C10—C9—H9	119.8
O1—C1—N1	122.3 (2)	C11—C10—C9	120.2 (3)
O1—C1—C2	120.8 (2)	C11—C10—H10	119.9
N1—C1—C2	116.9 (2)	C9—C10—H10	119.9
C3—C2—C7	119.1 (2)	C10—C11—C12	120.4 (3)
C3—C2—C1	117.4 (2)	C10—C11—H11	119.8
C7—C2—C1	123.4 (2)	C12—C11—H11	119.8
C4—C3—C2	120.6 (2)	C11—C12—C17	121.8 (3)
C4—C3—H3	119.7	C11—C12—C13	119.8 (3)
C2—C3—H3	119.7	C17—C12—C13	118.3 (3)
C3—C4—C5	120.1 (3)	C8—C13—C12	117.9 (2)
C3—C4—H4	120.0	C8—C13—C14	123.9 (2)
C5—C4—H4	120.0	C12—C13—C14	118.2 (2)
C6—C5—C4	119.8 (3)	C15—C14—C13	120.5 (3)
C6—C5—H5	120.1	C15—C14—H14	119.8
C4—C5—H5	120.1	C13—C14—H14	119.8
C5—C6—C7	120.1 (3)	C14—C15—C16	121.1 (3)
C5—C6—H6	119.9	C14—C15—H15	119.4
C7—C6—H6	119.9	C16—C15—H15	119.4
C2—C7—C6	120.2 (2)	C17—C16—C15	119.7 (3)
C2—C7—H7	119.9	C17—C16—H16	120.1
C6—C7—H7	119.9	C15—C16—H16	120.1
C9—C8—C13	121.1 (2)	C16—C17—C12	122.1 (3)
C9—C8—N1	118.6 (2)	C16—C17—H17	119.0
C13—C8—N1	120.2 (2)	C12—C17—H17	119.0
			
C8—N1—C1—O1	−6.5 (4)	C9—C10—C11—C12	2.2 (4)
C8—N1—C1—C2	171.9 (2)	C10—C11—C12—C17	178.7 (3)
O1—C1—C2—C3	24.1 (3)	C10—C11—C12—C13	0.1 (4)
N1—C1—C2—C3	−154.3 (2)	C9—C8—C13—C12	5.4 (3)
O1—C1—C2—C7	−156.2 (2)	N1—C8—C13—C12	−176.3 (2)
N1—C1—C2—C7	25.4 (3)	C9—C8—C13—C14	−174.6 (2)
C7—C2—C3—C4	−1.1 (3)	N1—C8—C13—C14	3.7 (3)
C1—C2—C3—C4	178.6 (2)	C11—C12—C13—C8	−3.8 (4)
C2—C3—C4—C5	1.6 (4)	C17—C12—C13—C8	177.6 (2)
C3—C4—C5—C6	−0.4 (4)	C11—C12—C13—C14	176.2 (2)
C4—C5—C6—C7	−1.2 (4)	C17—C12—C13—C14	−2.4 (4)
C3—C2—C7—C6	−0.5 (4)	C8—C13—C14—C15	−178.5 (2)
C1—C2—C7—C6	179.8 (2)	C12—C13—C14—C15	1.5 (4)
C5—C6—C7—C2	1.7 (4)	C13—C14—C15—C16	1.0 (4)
C1—N1—C8—C9	−117.0 (3)	C14—C15—C16—C17	−2.6 (5)
C1—N1—C8—C13	64.6 (3)	C15—C16—C17—C12	1.5 (5)
C13—C8—C9—C10	−3.2 (4)	C11—C12—C17—C16	−177.6 (3)
N1—C8—C9—C10	178.4 (2)	C13—C12—C17—C16	1.0 (5)
C8—C9—C10—C11	−0.7 (4)		

### Hydrogen-bond geometry (Å, °)

**Table:**

*D*—H···*A*	*D*—H	H···*A*	*D*···*A*	*D*—H···*A*
N1—H1···O1^i^	0.86	2.21	2.892 (3)	136.

Symmetry codes: (i) −*x*+1/2, *y*+1/2, *z*.

## References

[bb1] Allen, F. H., Kennard, O., Watson, D. G., Brammer, L., Orpen, A. G. & Taylor, R. (1987). *J. Chem. Soc. Perkin Trans. 2*, pp. S1–19.

[bb2] Bruker (2007). *SMART*, *SAINT* and *SADABS* Bruker AXS Inc., Madison, Wisconsin, USA.

[bb3] Sheldrick, G. M. (2008). *Acta Cryst.* A**64**, 112–122. 10.1107/S0108767307043930 18156677

[bb4] Zhang, S., Zhang, Y., Wang, C. & Zhu, R. (2011). *Acta Cryst.* E**67**, o2831. 10.1107/S1600536811039201 PMC3247570 22219875

